# Geographical variation and clustering are found in atrial fibrillation beyond socioeconomic differences: a Danish cohort study, 1987–2015

**DOI:** 10.1186/s12942-021-00264-2

**Published:** 2021-03-01

**Authors:** Kirstine Wodschow, Kristine Bihrmann, Mogens Lytken Larsen, Gunnar Gislason, Annette Kjær Ersbøll

**Affiliations:** 1 National Institute of Public Health, University of Southern Denmark, Studiestræde 6, 1455 Copenhagen K, Denmark; 2 Department of Clinical Medicine, Aalborg University, Aalborg, Denmark; 3 Department of Cardiology, Herlev and Gentofte Hospital, Herlev, Denmark; 4 Faculty of Health and Medical Sciences, University of Copenhagen, Copenhagen, Denmark; 5 The Danish Heart Foundation, Copenhagen, Denmark

**Keywords:** Atrial fibrillation, Epidemiology, Cluster analysis, Health status disparities, Health registers, Bayesian analysis.

## Abstract

**Background:**

The prevalence and incidence rate of atrial fibrillation (AF) increase worldwide and AF is a risk factor for more adverse cardiovascular diseases including stroke. Approximately 44% of AF cases cannot be explained by common individual risk factors and risk might therefore also be related to the environment. By studying geographical variation and clustering in risk of incident AF adjusted for socioeconomic position at an individual level, potential neighbourhood risk factors could be revealed.

**Methods:**

Initially, yearly AF incidence rates 1987–2015 were estimated overall and stratified by income in a register-based cohort study. To examine geographical variation and clustering in AF, we used both spatial scan statistics and a hierarchical Bayesian Poisson regression analysis of AF incidence rates with random effect of municipalities (n = 98) in Denmark in 2011–2015.

**Results:**

The 1987–2015 cohort included 5,453,639 individuals whereof 369,800 were diagnosed with an incident AF. AF incidence rate increased from 174 to 576 per 100,000 person-years from 1987 to 2015. Inequality in AF incidence rate ratio between highest and lowest income groups increased from 23% in 1987 to 38% in 2015. We found clustering and geographical variation in AF incidence rates, with incidence rates at municipality level being up to 34% higher than the country mean after adjusting for socioeconomic position.

**Conclusions:**

Geographical variations and clustering in AF incidence rates exist. Compared to previous studies from Alberta, Canada and the United States, we show that geographical variations exist in a country with free access to healthcare and even when accounting for socioeconomic differences at an individual level. An increasing social inequality in AF was seen from 1987 to 2015. Therefore, when planning prevention strategies, attention to individuals with low income should be given. Further studies focusing on identification of neighbourhood risk factors for AF are needed.

## Background

Atrial fibrillation (AF) is the most common heart rhythm disorder (arrhythmia) and represents a global epidemic [1–3] with a worldwide increase in both age adjusted prevalence and incidence rates (IRs) [2, 4]. AF is a risk factor for more severe conditions, including stroke [5]. In 2010, 33.5 million individuals were estimated to be living with AF worldwide and 8.8 million adults in Europe alone [6]. In Denmark, the AF IR increased from 98 to 307 per 100,000 person-years from 1983 to 2012 [7].

Individual-level AF risk factors are well described [8–11], e.g. advancing age, male sex, obesity, prior cardiac disease, and elevated blood pressure. However, it has been estimated that 44% of the variation in risk of AF cannot be explained by these risk factors [12]. Low socioeconomic position has been associated with AF [13], though the findings are still not conclusive [14, 15]. Risk of and differences in incident AF might be associated with environmental and societal neighbourhood differences. A Canadian study [16] found geographical variation in several emergency department presentations for AF, which they suggest may be explained by the possibility of higher disease severity in some areas or different availability of non-emergency department health services. Furthermore, geographical variation in AF hospitalization rates were found between states in the United States [17]. However, none of the two studies included socioeconomic position in the analysis. By studying geographical variation in risk of cardiovascular diseases potential neighbourhood risk factors could be revealed [18] e.g. related to the physical or social environment [19] and further attention could be given to areas with high risk of AF.

The aim of this study was to examine geographical variation and clustering in AF IRs adjusted for socioeconomic differences.

The objectives were to: [1] initially, examine developments in annual AF IRs from 1987 to 2015 stratified by income, and [2] analyse if geographical variation and clusters exist in risk of incident AF in 2011–2015, adjusted for age, sex and socioeconomic position.

## Methods

### Study design and study area

We conducted a cohort study based on prospectively collected individual-level data from Danish nationwide registers. Registers were linked at individual-level using the unique personal identification number assigned to each Danish citizen at birth or immigration [20]. In Denmark, healthcare (such as general practitioner and hospitals) is financed through taxes and thereby free of charge. The study area covers Denmark, approximately 43,000 km^2^ divided into 98 municipalities. Until 2007, Denmark was divided into 275 municipalities.

### Population

An open cohort of individuals age ≥ 30 years at inclusion, with residential location in Denmark and with no previous AF diagnosis was created. The study period was 1987–2015. Individuals were followed until date of first AF diagnosis, death, emigration or end of study (December 31, 2015), whichever came first. No re-entry was allowed after censoring. For the geographical analyses, the study period was restricted to 2011–2015, since the focus was to identify the most present geographical variations. For details on how registers were linked and data workflow on how the two cohorts (1987–2015 and 2011–2015) were derived see Additional file 1.

### Atrial fibrillation

Incident AF was defined as first atrial flutter or AF diagnosis (primary or secondary diagnosis) in the Danish National Patient Register (NPR) [21], including both inpatient and outpatient data (after 1995 outpatient speciality clinic diagnoses were included [22]), or AF (including atrial flutter) as a cause of death extracted from the Danish Register of Causes of Death (DRCD) (including underlying and contributory causes), whichever came first. In accordance with similar epidemiological studies [2, 14, 15], the less common atrial flutter diagnosis was included. Diagnoses were classified according to the International Classification of Disease, 8th revision (ICD-8) from 1977 to 1993 (codes 427.93, 427.94 in NPR and 427.9 in DRCD) and the 10th revision (ICD-10) thereafter (codes I48). The positive predictive value of AF in NPR has been estimated to 92.6% [23].

### Individual-level characteristics

Annually registered individual-level data on sex, date of birth, cohabitation, residential address and residential municipality (according to both pre- and post-2007 municipality definition, (n = 275 and n = 98, respectively)) were obtained from the Danish Civil Registration System [20]. Individuals were categorized in four age groups (30–59, 60–69, 70–79 and ≥ 80 years) for each calendar year. As a proxy for health behaviour and lifestyle (such as smoking and alcohol consumption not available in the registers) socioeconomic position was used. The three variables cohabitation, education, and income were used to describe socioeconomic position. Cohabitation was defined as a binary variable (married or living with a partner, single). Family equivalent income at an individual level was obtained from the Income Register [24] and calculated based on personal income (1987–1989) and annually registered individual-level family equivalent household income (1990–2015). A one-year offset was used since income was registered on the last day of the year. Income was categorized in quintiles within calendar year, age (< 65 years, ≥ 65 years) and sex. Educational level at individual-level was obtained from the Education Register [25] and categorized in 3 groups (elementary: ≤ 9 years, short: 10–12 years and medium/long: ≥ 13 years). Missing educational level was replaced by previous or following (if previous was missing) educational level. When educational level was registered as unknown (5.3% of the study population (2011–2015)), the lowest educational level was assigned. In the cohort, individuals with missing educational level mainly belonged to the lowest income group.

### Statistical analysis

Temporal development in AF IRs (objective 1) was examined by calculating yearly IRs for 1987–2015 stratified by income. Wald test was used to test if the relative difference between lowest and highest income groups changed from 1987 to 2015.

Objective 2 examines if geographical variation and clustering in incident AF exist. Geographical variation is used as a more general term to describe if the incidence of AF in some areas (e.g. municipalities) is higher or lower compared to the country mean. Clustering is used to identify specific areas where the risk within the area (e.g. group of neighboring municipalities or group of individuals) is higher (or lower) than outside the area. First, a descriptive analysis of clustering in AF risk with residential addresses as the geographical unit was performed. However, due to privacy concerns it is not possible to include socioeconomic position in this analysis. Therefore, geographical variation and clustering in AF IRs at municipality level were analysed afterwards. In these analyses we were able to adjust for socioeconomic position. The specific methods applied are described below.

A cluster analysis of incident AF with residential addresses as the geographical unit was performed to identify significant local high-risk clusters of AF (2011–2015). We used spatial scan statistics with residential addresses as the geographical unit and a Bernoulli probability model to evaluate significance (*p* value < 0.05) and approximate location of clusters [26]. Analyses were stratified by age group. A circular search window and 999 Monte Carlo replications were applied in the analyses. Clusters where the centre did not overlap with more significant clusters were reported. For individuals with more than one address in 2011–2015, the address on which the person lived the longest was used. Different search windows were applied: including up to 5% or 10% of the population, or a maximum distance of 5 km or 10 km. By changing the search window, we were able to detect clusters at municipality level and at smaller scale, not detectable in the following regression analysis.

Afterwards, we investigated geographical variation and clustering in AF IRs in the period 2011–2015 with municipalities (n = 98) as geographical unit and with adjustment for individual risk factors (age, sex and socioeconomic position). We did a hierarchical Bayesian Poisson regression analysis with number of incident AF cases as outcome and logarithmic transformation of follow-up time as offset (piecewise exponential model) [27]. Follow-up time was split based on calendar year and age group.

Individuals, and thereby also risk of disease, within a given area tend to be more similar than individuals further away [28]. By using a hierarchical model with random effects of municipality, we could account for correlation within and between municipalities as well as fixed effects of individual-level risk factors [29]. The random effect is an estimate of the residual IR ratios between each municipality and the country mean after accounting for individual-level risk factors.

Initially, a semi-adjusted model including age, sex and random effect of municipality was applied. Next, a fully adjusted model with additional adjustment for socioeconomic position given by income, education and cohabitation at individual level was applied. No adjustment for calendar year was performed due to computational problems caused be the size of the dataset. However, the yearly IRs in 2011 to 2015 were similar, and an adjustment for calendar year is therefore expected to have less influence on the results. The random effect of municipality was spilt into the sum of an unstructured component and a geographically structured component, usually referred to as the BYM model [30]. The unstructured components were modelled as independent and identically Gaussian distributed (IID). The geographically structured components were modelled by a conditional autoregressive (CAR) model [30] based on a binary 98 × 98 adjacency matrix, where 1 indicates municipalities sharing the same border. Islands were linked to municipalities according to main transport route by ferry or bridge. (For further details on the model see Additional file 2).

Bayesian inference (parameter estimation) was performed by Integrated Nested Laplace Approximation (INLA) [31]. For the fixed effects, a default Gaussian (0,0.001) prior distribution was assigned. The precision parameter of the IID components and the hyper parameter of the CAR model were both assigned a log-gamma prior distribution with default parameters (1,0.0005). To evaluate the effect of the prior distributions, a sensitivity analysis was performed by changing the log-gamma parameters to (1,0.05) and (1,0.000005) in the fully adjusted model. Estimates were reported as the mean with 95% credible interval based on the posterior distribution. Estimated random effects were mapped as residual IR ratios for each municipality compared to the country mean.

To evaluate the presence of residual spatial autocorrelation between municipalities, the BYM model was compared to a model with the geographically structured random effect component excluded. Models were compared using the Bayesian Deviance Information Criterion (DIC) [32], where smaller values indicate a better fit to data. In the BYM model, fraction of spatial variation out of the total variation was also calculated.

Three supplementary Poisson regression analyses were performed. To investigate the effect of a higher spatial resolution (i.e. smaller geographical units) on the geographical variation and clustering in incident AF, the fully adjusted model was applied to data with pre-2007 defined municipalities (n = 275) as geographical unit. To investigate the possible misclassification of educational level, an analysis including only those individuals with registered educational level was performed. Finally, to evaluate the effect of hypertension, a large risk factor for AF, an analysis with additional adjustment for hypertension was performed. Hypertension was a binary variable (yes, no) defined based on prescription redemptions of antihypertensive drugs. Having at least two of the following classes of antihypertensive drugs within a year defined hypertension: α adrenergic blockers, non-loop diuretics, vasodilators, β blockers, calcium channel blockers and renin-angiotensin system inhibitors [33].

We used STATA statistical software version 15.1 (Stata College Station, TX) for data management, and calculation of IRs and IR ratios. The INLA package (www.r-inla.org) was used in R x64 V.3.5.1 for Bayesian hierarchical Poisson regression, and spatial scan statistics were performed in SaTScan version 9.6 (www.satscan.org). Quantum GIS version 3.2.1. (www.qgis.org) was used for visualisation of geographical variation and clusters.

## Results

**Fig. 1:**
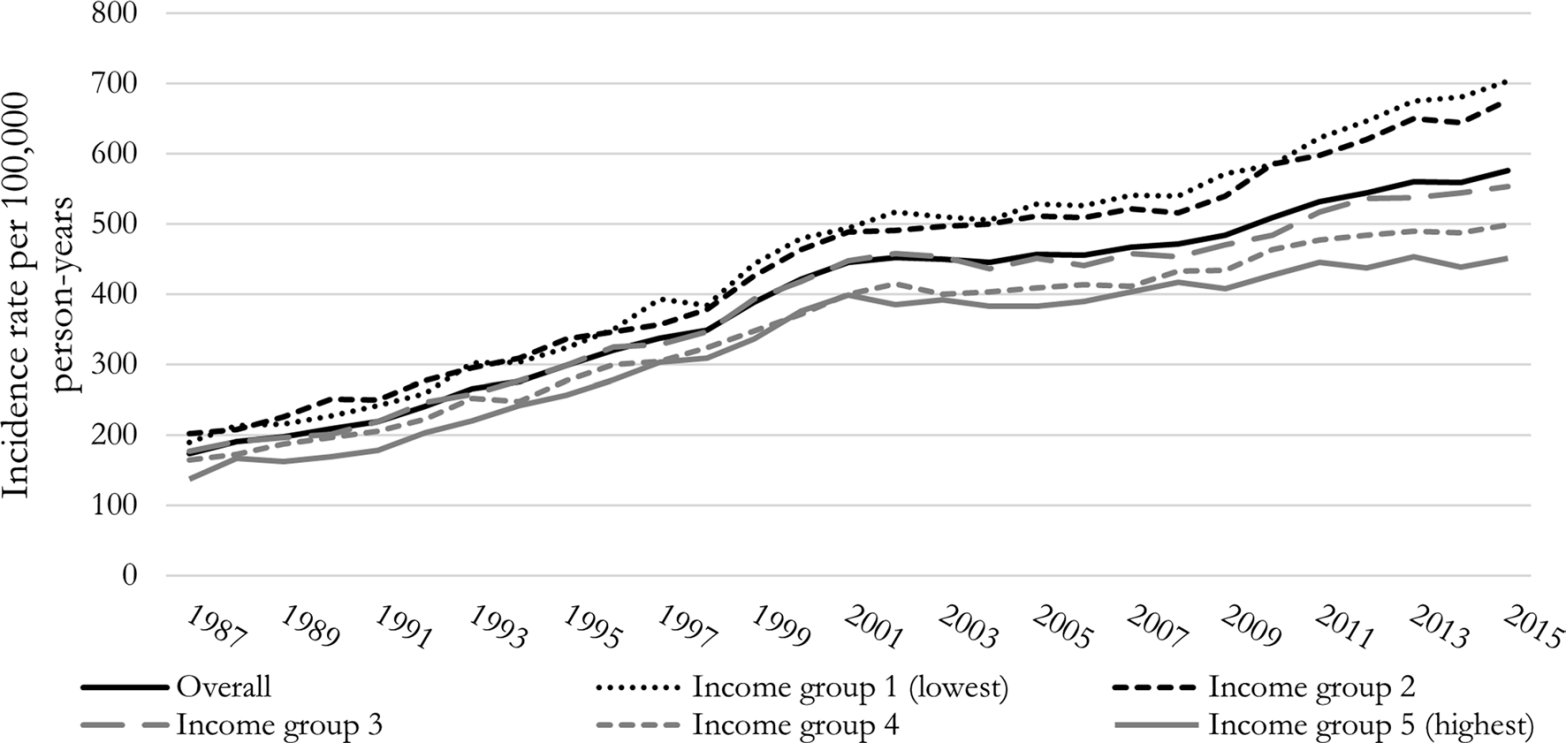
Annual atrial fibrillation (AF) (including atrial flutter) incidence rate per 100,000 person-years from 1987 to 2015 in Denmark, overall and stratified by income groups

A total of 5,453,639 individuals (93,854,020 person-years) were included in the 1987–2015 cohort, whereof 369,800 were diagnosed with incident AF (IR = 393.7 per 100,000 person-years). Annual overall and income stratified IRs (1987–2015) are shown in Fig. 1. The overall IR increased from 174.1 to 576.0 per 100,000 person-years from 1987 to 2015 (95% CI 169.4–178.8; 567.9–584.1). The difference in IR between lowest and highest income groups increased from 52.6 to 252.8 per 100,000-person years from 1987 to 2015 (95% CI 38.35–67.21; 227.19–278.32). The IR ratio between the lowest and highest income group increased significantly from 1.39 to 1987 to 1.56 in 2015 (95% CI 1.27–1.51; 1.49–1.63, *p* value = 0.020).

In the subset of the cohort (2011–2015), used in the geographical analyses at municipality level, 3,736,883 individuals (16,850,154 person-years) were included whereof 93,434 had an incident AF (IR = 554.1 per 100,000 person-years). Baseline characteristics at inclusion (Table 1) shows that most of the individuals were 30–59 years (68%) and married or living with a partner (67%). Nearly half had 10–12 years education (45%) while the individuals were close to evenly distributed in the five income groups (categorization of income was based on quintiles). The AF IR was 616.8 and 495.8 per 100,000 person-years among men and women, respectively (Table 1), and increased from 111.1 to 3,346.3 per 100,000 person-years from the youngest (30–59 years) to the oldest age group (≥ 80 years).

**Table 1:** Baseline characteristics at date of inclusion of adults ≥ 30 years with a residential address in Denmark 2011–2015 and characteristics of individuals with incident atrial fibrillation (AF) (including atrial flutter) on date of diagnosis (2011–2015). Incidence rate (IR) and 95% confidence interval (CI)

		Study population at baseline		Incidence of atrial fibrillation (2011–2015)			
		N = 3,736,883	%	N = 93,434	%	IR per 100,000 person-years	95% CI
Sex							
Men		1,814,936	48.6	50,135	53.7	616.8	611.5-622.3
Women		1,921,947	51.4	43,299	46.3	495.8	491.1-500.4
Age							
30–59		2,553,879	68.3	11,966	12.8	111.1	109.1-113.1
60–69		642,007	17.28	20,746	22.2	645.4	636.7-654.3
70–79		349,286	9.4	28,466	30.5	1487.8	1470.6-1505.2
≥ 80		191,711	5.1	32,256	34.5	3346.3	3310.0-3383.0
Education							
≤ 9 years		1,179,760	31.6	44,631	47.8	894.5	886.2-902.8
10–12 years		1,672,527	44.86	35,107	37.6	452.5	447.8-457.2
≥ 13 years		884,596	23.7	13,696	14.7	333.0	327.5-338.6
Income							
Lowest		827,329	22.1	22,199	23.8	665.7	657.0-674.6
Second lowest		749,959	20.1	21,418	22.9	637.7	629.2-646.3
Middle		734,984	19.7	18,134	19.4	537.5	529.7-545.4
Second highest		719,943	19.3	16,520	17.7	487.3	480.0-494.8
Highest		704,668	18.9	15,163	16.2	445.3	438.3-452.5
Cohabitation							
Married or living with a partner		2,508,211	67.1	49,745	53.2	434.1	430.3-437.9
Single		1,228,672	32.9	43,689	46.8	808.7	801.1-816.3

N, number of persons

IR, incidence rate

CI, confidence interval

**Fig. 2:**
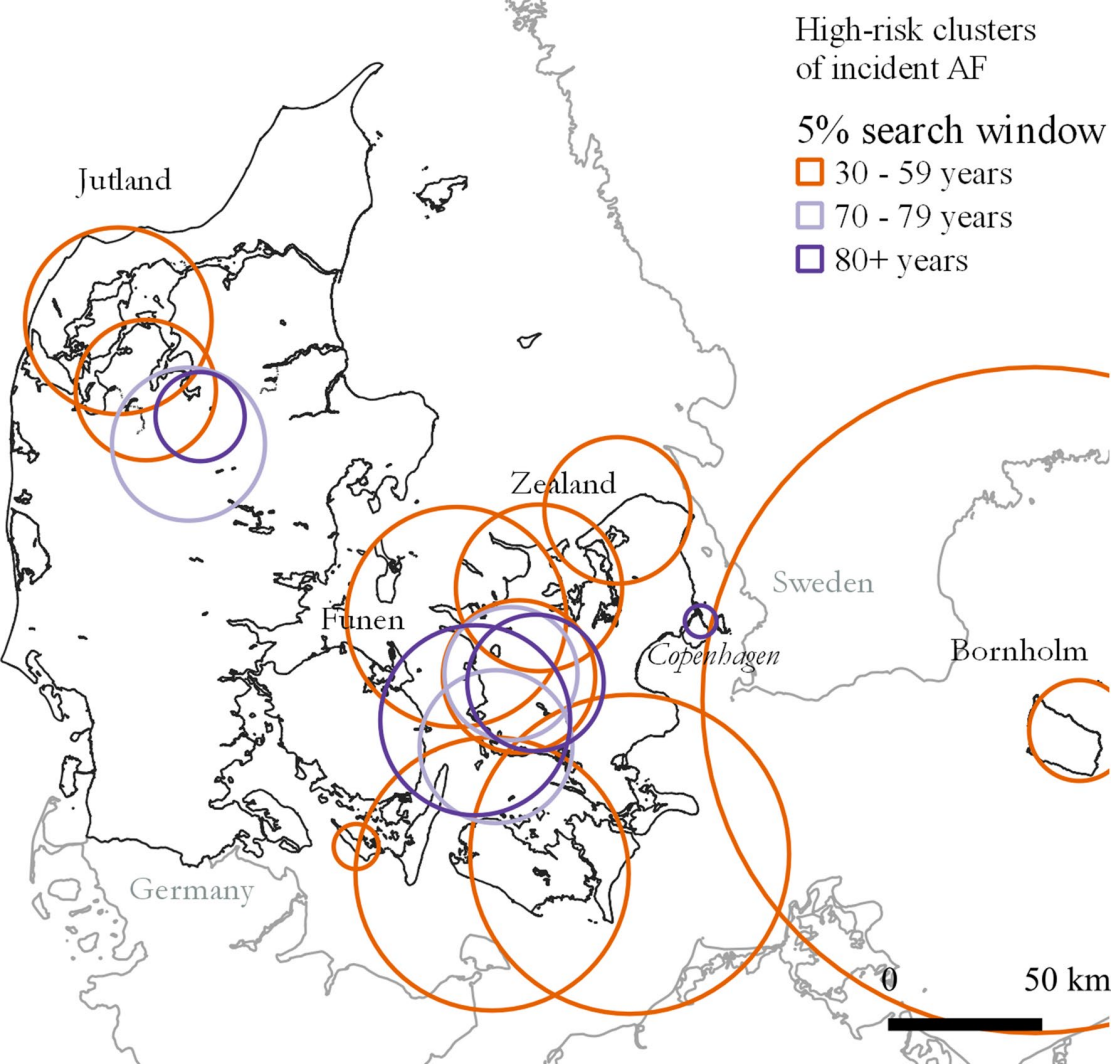
High-risk clusters of incident atrial fibrillation (including atrial flutter) stratified by age (year 2011–2015). Only statistically significant cluster are shown (*p* value < 0.05). Map contain data from © EuroGeographics for the administrative boundaries

Results of the initial descriptive analysis of AF risk with residential addresses as the geographical unit are described in the following. A map showing local clusters in incident AF (2011–2015) stratified on age groups and with residential addresses as the geographical unit is presented in Fig. 2. A slightly higher number of individuals (n = 3,733,246 and 95,950 incident AF) were included compared to the number of individuals in the geographical analyses at municipality level. For details see Additional file 3. Clusters with a significantly higher AF risk inside compared to outside of clusters were detected for age groups 30–59, 70–79 and ≥ 80 years. The highest number of clusters (n = 11) were found for age group 30–59 years and no significant clusters were found in age group 60–69 years. Cluster radii ranged from 10.0 to 137.3, 28.1–77.8 and 1.4–173.5 km for age groups 30–59, 70–79 and ≥ 80 years, respectively (Additional file 4). The large circle around Bornholm include a few individuals in the area of Copenhagen.

In the hierarchical Bayesian Poisson regression analysis, the BYM model provided a better fit to data than the model with the geographically structured random effect component excluded (semi-adjusted: DIC = 79,527 vs. DIC = 79,534; fully adjusted: DIC = 78,109 DIC = 78,118). This indicates that there is residual spatial autocorrelation between neighbouring municipalities. In the following, results are only presented for the BYM models.

**Fig. 3:**
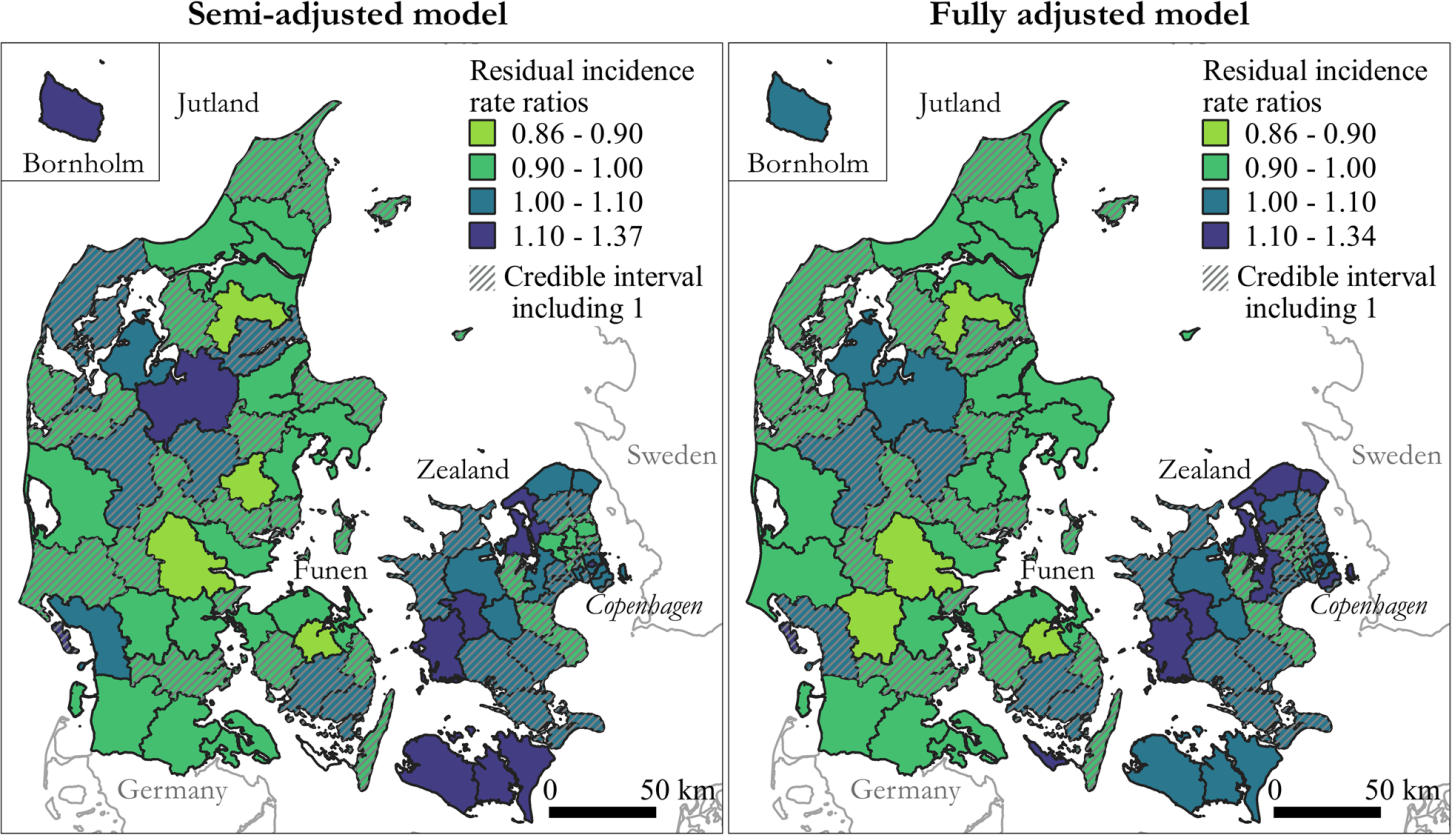
Geographical distribution of the residual incidence rate (IR) ratios of atrial fibrillation (AF) (including atrial flutter) at municipality level compared with country mean (2011–2015) for the semi-adjusted model, adjusted for age and sex and random effect of municipality, and the fully adjusted model, additionally adjusted for socioeconomic position. Municipalities where the residual AF IR is not significantly different from the country mean (i.e. the 95% credibility interval include 1) are grey shaded. Maps contain data from © EuroGeographics for the administrative boundaries and from The Danish Agency for Data Supply and Efficiency, municipality borders, 2019

Maps showing geographical variation in residual AF IRs (2011–2015) for the 98 municipalities compared to the country mean are presented in Fig. 3 for the semi-adjusted and the fully adjusted model. Residual IR ratios is a measure of the estimated AF risk within each municipality compared to the country mean, after adjusting for age and sex (semi-adjusted model) and socioeconomic position (fully adjusted model). Clusters of high AF residual IR ratios (semi-adjusted model in Fig. 3) were mainly found on Zealand, smaller islands, and the northern part of central Jutland. Location of clusters were similar to the location of clusters found in the descriptive analysis with residential addresses as the geographical unit. Adjusting for socioeconomic position resulted in less uncertainty on the estimates, but the reduction in the geographical variation was minimal. However, the residual IR ratios increased in the northern municipalities of Zealand. Residual IRs at municipality level were up to 34% higher than the country mean after adjusting for socioeconomic position. The geographical pattern remained with higher residual IR ratios in the northern and western part of Zealand and the northern part of central Jutland (Fig. 3). In the fully adjusted model, the fraction of spatial variation was 0.32. This confirmed the presence of residual spatial autocorrelation between neighbouring municipalities, although it was not the dominating source of unexplained variation. Changing the parameters of the prior distributions did not influence the results (see Additional file 5).

In the supplementary analysis of the geographical variation (2011–2015) with pre-2007 definition of municipalities (n = 275) as geographical unit, 38,400 individuals (whereof 533 had an incident AF) were excluded due to missing data on information about municipality. The overall geographical pattern in residual IR ratios remained the same as for in main analysis (Additional file 5). However, due to less statistical power within each municipality the residual AF IR was not significantly different from the country mean for a larger number of municipalities. When excluding individuals with missing educational level the overall geographical variations remained (Additional file 5), however, the total IR decreased by 13/100,000 person-years. Adjusting for hypertension did not change the overall clusters of AF residual IR ratios, however, the upper limit of residual IR ratio increased from 1.34 to 1.48 (Additional file 5). IR among individuals with hypertension was 2.378 per 100.000 person-years.

## Discussion

### Summarizing results

In this nationwide register-based cohort study, we found that annual IRs of AF increased from 1987 to 2015 and highest IRs were associated with low income. Social inequality in the IR of AF increased during the study period. Geographical variation and clustering in AF IRs were seen even after adjusting for socioeconomic position given by cohabitation, education and income.

### Interpretation of results

The overall temporal variation in IRs is consistent with the global predictions of AF [1, 6]. As discussed by Schmidt et al. [7] and Frost al. [34] the increase in IR of AF from 1987 to 2015 might be explained by e.g. an enhanced detection, changed diagnostic criteria and an increased prevalence of risk factors for AF (such as high body mass index and hypertension).

The increased socioeconomic inequality in AF might partly be explained by an increase in the prevalence of risk factors in the lowest compared to the highest income groups, since low socioeconomic position is associated with higher risk for AF risk factors e.g. type 2 diabetes mellitus [35] and heart failure [36]. From a public health perspective, it is worth noticing, that low socioeconomic position is associated with higher mortality in AF patients [37, 38].

The geographical variation in AF IR ratios when adjusted for socioeconomic position supports the earlier findings in Alberta, Canada [16], where clusters in emergency department presentations for AF were found. We augment their findings, by showing that clusters in AF IR ratios also exist at a smaller geographical scale and when adjusting for socioeconomic position.

The municipalities with higher residual AF IRs compared to the country mean share no obvious similarities. Both less populated (e.g. Bornholm) and highly populated (Copenhagen) municipalities have an increased AF IR compared to the country mean even after adjusting for socioeconomic position. Interestingly, when adjusting for socioeconomic position, the AF residual IR ratios increased in the northern municipalities of Zealand, which is an area with higher income than the country mean [39]. The higher AF residual IR ratios in the western part of Zealand and the islands south of Zealand, where the income is lower than the country mean [39], is similar to what was found by Kjærulff et al. [40] for risk of acute myocardial infarction (AMI) in Denmark. The area with higher residual IR ratios in northern mid-Jutland was not found for AMI. The different results in the studies indicate that underlying risk factors are to some extent geographically different for the two cardiovascular diseases.

Several local clusters of high AF risk were overlapping for the four age groups. Similar findings were reported by Naderi et al. [17] where the AF hospitalization rates were higher than the national average for both young (< 65 years) and older (≥ 65 years) patients in several of the same states. However, when considered at a regional scale, they found differences in how likely old versus young individuals were to be hospitalized compared to the national average. The highest number of clusters of AF risk were detected for age group 30–59 years, and the location of these clusters corresponded to the location of areas with high residual IR ratios found in the regression analysis without adjusting for socioeconomic position. This may indicate, that socioeconomic position is stronger associated with AF in the youngest age group, as has been shown by Lunde et al. for educational level [13]. However, when increasing the search window to include 10% of the population, statistically significant clusters were detected in age groups 70–79 and ≥ 80 years in the same areas as for age group 30–59 years. For age group 60–69, AF risk may be more randomly distributed compared to the other age groups, since no significant clusters were detected for this group.

Possible geographically dependent factors that might explain some of the geographical variation in AF IRs include e.g. regional differences in the healthcare system, accessibility and distance to the general practitioner or environmental exposure. A previous study showed that contact to the general practitioner prior to AMI was less frequent in Northern Jutland compared to the rest of Denmark [41]. Less contact to the general practitioner may result in underestimated AF IR in the northern municipalities in Jutland. Air pollution might be a possible environmental risk factor for AF, since an 8% higher risk of AF was found among adults in Denmark for an increase of 10 µg/m^3^ NO_2_ [42]. Furthermore, the geographical variation in small particulate matter (PM_2.5_) in Denmark [43] is to some extent similar to the geographical variation in AF IRs found in the present study. However, both a positive association [44] and no association [45, 46] between AF and PM_2.5_ have been found.

### Strengths and limitations

Strengths of the study include a large population size that strengthens the accuracy of the results, the use of nearly the entire Danish adult population, a high positive predictive value of AF [23] and individual-level linkage of data. Furthermore, to our knowledge this was the first study on geographical variation and clustering in incident AF, with adjustment for socioeconomic position at an individual level.

Incident AF is biased towards more severe cases, since unrecognized AF is common and many AF patients remain asymptomatic, [2, 47] which might lead to underestimated IRs. Left truncation bias may occur, since NPR only dates back to 1977. The risk of being incorrectly counted as incident is higher in the beginning of the study period compared to the end of the period, which might have resulted in a slightly overestimated IR in the beginning of the study period. However, due to changes in awareness of AF, and changes in diagnostic practice it is more likely that the IRs in the beginning of the study period are underestimated. No distinction was made between different types of AF (paroxysmal, persistent or long-term persistent) and atrial fibrillation versus atrial flutter. It is likely that the risk factors deviate between the different diagnoses.

A common limitation in register-based studies is the lack of controlling for unmeasured confounders [48]. Although we have adjusted for age, sex and socioeconomic position, it is possible that some of the geographical variation in AF could be explained by individual risk factors, e.g. alcohol and hypertension. However, after adjusting for hypertension, the overall clusters of AF IR ratios remained. Alcohol has only partly been accounted for by including socioeconomic position. Geographical variation in alcohol consumption in Denmark is seen, however, information is only available for a limited subset of the population [49].

The geographical variation in risk of AF depends on scale and boundary of the selected geographical unit [50]. Variation in the cultural and structural neighbourhood or geographical variation in potential environmental exposures smaller than the size of the municipalities, cannot be detected when municipalities are the geographical unit. However, the clusters identified in the analysis using residential addresses correspond to the geographical variation in AF IRs found in the analysis with municipalities as the geographical unit adjusted for age and sex. This indicates that even small clusters in AF risk were detected at municipality level and that municipality level is an appropriate scale to detect both clusters and geographical variations in AF risk. Furthermore, the fully adjusted model was applied to the former 275 municipalities, where the same high-IR areas were detected. Due to restricted access to socioeconomic data, we were not able to adjust for socioeconomic position in the local cluster analyses with residential addresses as geographical unit, which limited the comparison with the results on geographical variation in AF residual IRs from the regression analyses.

## Conclusions

Our study extent current knowledge by showing an increasing social inequality in incident AF in 1987–2015. Geographical variation in incident AF was seen in a study population with free healthcare and remained after adjusting for socioeconomic position. Notably, after adjusting for socioeconomic position geographical areas with higher socioeconomic position had significantly higher incidence of AF. Furthermore, municipalities with either higher or lower residual IRs clustered. The geographical variation and clustering in incident AF were consistent when using smaller geographical units. In future prevention strategies, attention should be given to individuals with low income. Further studies focusing on identification of neighbourhood risk factors are needed. We suggest investigating if geographical variations in PM_2.5_ or location and availability of primary and secondary health services might explain some of the geographical variation and clustering in incident AF.

## Supplementary Information

**Additional file 1.** Flow chart of data with indication of numbers (n) of individuals censored at each step in the data management and numbers of incident atrial fibrillation (AF).

**Additional file 2.** Statistical model.

**Additional file 3.** Origin of register data and overview of cohorts and analyses used in the study.

**Additional file 4.** Detected clusters of high-risk atrial fibrillation stratified by age and for four different search windows from the scan statistics analysis.

**Additional file 5.** Sensitivity analysis and supplementary analyses.

## Data Availability

The data that support the findings of this study are available from the National Health Authority and Statistics Denmark, but restrictions apply to the availability of these data, which were used under license for the current study, and so are not publicly available.

## Abbreviations

AF: Atrial fibrillation
AMI: Acute myocardial infarction
CI: Confidence interval
DIC: Deviance information criterion
DRCD: Danish register of causes of death
ICD: International classification of disease
IID: Independent and identically Gaussian distributed
INLA: Integrated nested laplace approximation
IR: Incidence rate
NPR: Danish National Patient Register
PM: Particulate matter
