# Supplementary material for: Geographical variation and clustering are found in atrial fibrillation beyond socioeconomic differences: a Danish cohort study, 1987–2015
Source: Int J Health Geogr. 2021 Mar 1;20:11. doi: 10.1186/s12942-021-00264-2 (PMC7923319; doi:10.1186/s12942-021-00264-2)

# Additional File 1

Supplementary material Additional File 1 for article “Geographical variation and clustering are found in atrial fibrillation beyond socioeconomic differences: A Danish cohort study, 1987-2015”

Additional File 1: Flow chart of data with indication of numbers (n) of individuals censored at each step in the data management and numbers of incident atrial fibrillation (AF). Access to the registers were obtained through secure servers at Statistics Denmark.


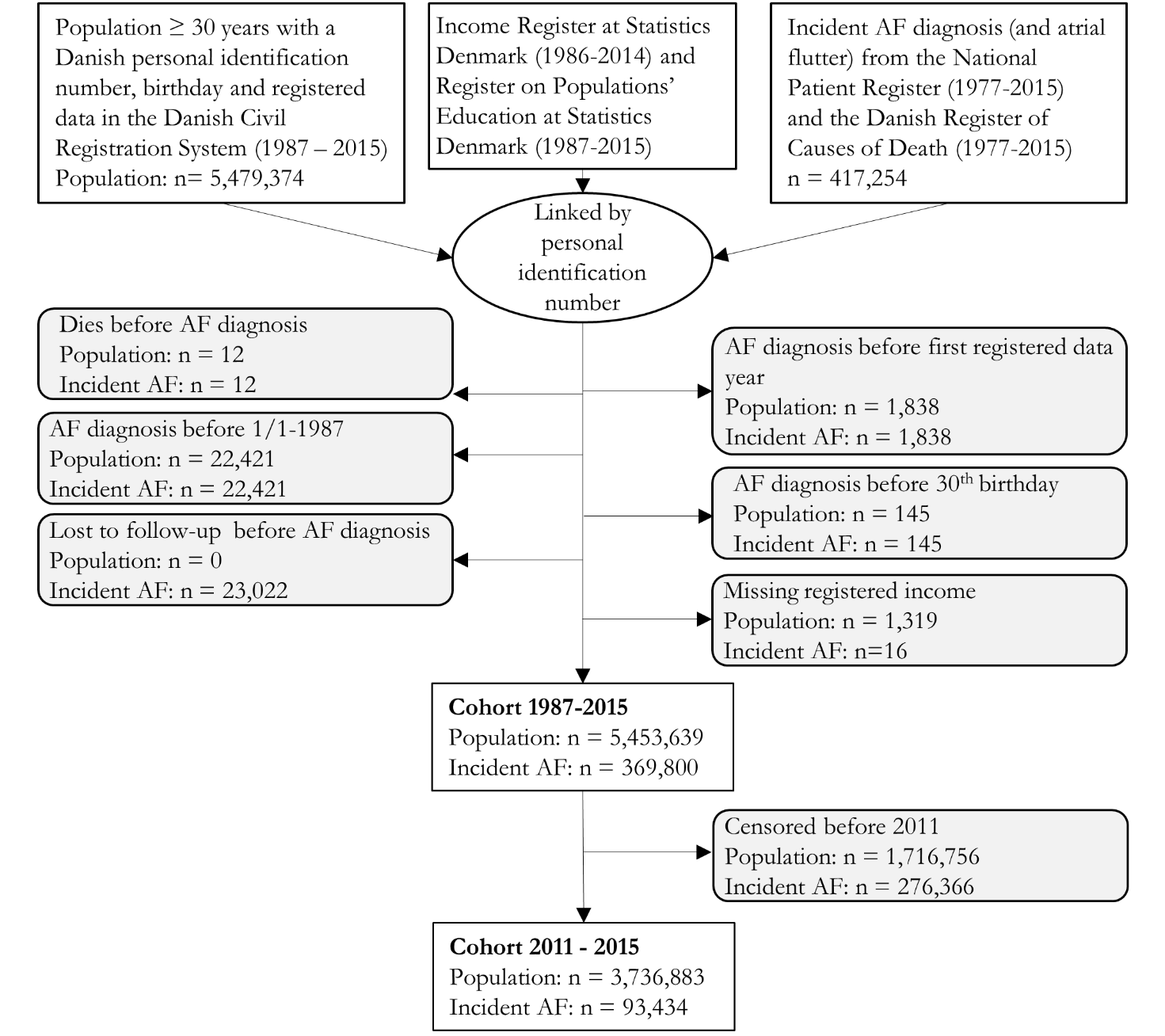

Supplement: Supplementary file 1 — Additional file 1. Flow chart of data with indication of numbers (n) of individuals censored at each step in the data management and numbers of incident atrial fibrillation (AF). [file 12942_2021_264_MOESM1_ESM.docx]
