# Supplementary material for: Geographical variation and clustering are found in atrial fibrillation beyond socioeconomic differences: a Danish cohort study, 1987–2015
Source: Int J Health Geogr. 2021 Mar 1;20:11. doi: 10.1186/s12942-021-00264-2 (PMC7923319; doi:10.1186/s12942-021-00264-2)
Supplement: Supplementary file 2 — Additional file 2. Statistical model. [file 12942_2021_264_MOESM2_ESM.docx]

**Additional File 2**

Additional File 2 for article “Geographical variation and clustering are found in atrial fibrillation beyond socio-economic differences: A Danish cohort study, 1987-2015”

Additional File2: Statistical model

Let $y_{ij}$ denote the number of incident atrial fibrillation cases in subgroup $i$, $i=1,\ldots,N_{\mathrm{group}}$, within municipality $j$, $j=1,\ldots,98$, where subgroup $i$ is given by a covariate vector $\boldsymbol{X}_{i}$. Then $Y_{ij}$ follows a Poisson distribution with parameter $\zeta_{ij}=t_{ij}\lambda_{ij}$, where $\lambda_{ij}$ is the incidence rate in subgroup $i$ within municipality j, and $t_{ij}$ is the person-years at risk.

Now the hierarchical Poisson regression model is given by

$\log\left( \zeta_{ij} \right)=\log\left( t_{ij} \right)+\boldsymbol{\beta X}_{i}+\mu_{j}+ʋ_{j}$, $i=1,\ldots,N_{\mathrm{group}}$, $j=1,\ldots,98$,

where $\boldsymbol{\beta}$ is a vector of unknown regression parameters including an intercept term, $\mu_{j}$ is an unstructured random effect of municipality $j$ modelled as Gaussian $(0, \sigma^{2})$, and $ʋ_{j}$ is a geographically structured random effect of municipality $j$ modelled by a conditional autoregressive (CAR) model with $ʋ_{j}|\boldsymbol{ʋ}_{-j}$ being Gaussian distributed with mean

$$\sum_{k=1}^{98} \frac{w_{jk}ʋ_{k}}{w_{jk}}$$

and variance

$$\frac{\tau^{2}}{\sum_{k=1}^{98} w_{jk}}$$

Here $w_{jk}$, $j=1,\ldots,98$, $k=1,\ldots,98$, is an element of the 98 x 98 binary adjacency matrix.
