# Supplementary material for: Geographical variation and clustering are found in atrial fibrillation beyond socioeconomic differences: a Danish cohort study, 1987–2015
Source: Int J Health Geogr. 2021 Mar 1;20:11. doi: 10.1186/s12942-021-00264-2 (PMC7923319; doi:10.1186/s12942-021-00264-2)

**Additional File 3**

Additional File 3 for article “Geographical variation and clustering are found in atrial fibrillation beyond socio-economic differences: A Danish cohort study, 1987-2015”

Additional File 3 Origin of register data and overview of cohorts and analyses used in the study. Atrial fibrillation (AF), number (n).


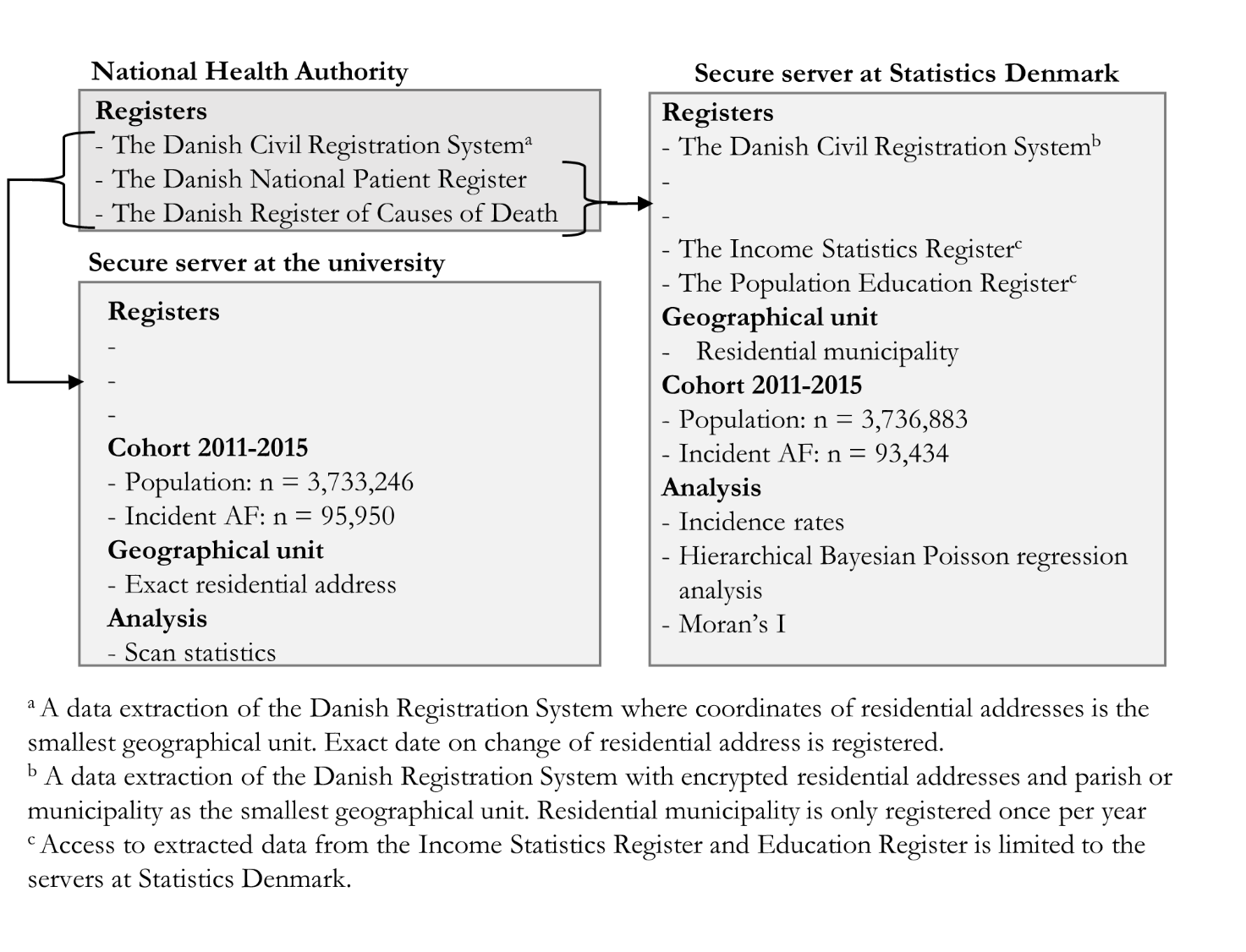

Supplement: Supplementary file 3 — Additional file 3. Origin of register data and overview of cohorts and analyses used in the study. [file 12942_2021_264_MOESM3_ESM.docx]
