# Supplementary material for: Geographical variation and clustering are found in atrial fibrillation beyond socioeconomic differences: a Danish cohort study, 1987–2015
Source: Int J Health Geogr. 2021 Mar 1;20:11. doi: 10.1186/s12942-021-00264-2 (PMC7923319; doi:10.1186/s12942-021-00264-2)
Supplement: Supplementary file 4 — Additional file 4. Detected clusters of high-risk atrial fibrillation stratified by age and for four different search windows from the scan statistics analysis. [file 12942_2021_264_MOESM4_ESM.docx]

**Additional File 4**

Additional File 4 for article “Geographical variation and clustering are found in atrial fibrillation beyond socio-economic differences: A Danish cohort study, 1987-2015”

Additional File 4: Detected clusters of high-risk atrial fibrillation stratified by age and for four different search windows from the scan statistics analysis.

| Age | Search window | Number of statistically significant cluster  (P-value <0.05) | Range of cluster radii [km] |
| --- | --- | --- | --- |
| 30-59 | 5 % | 11 | 10.0 – 137.3 |
|  | 10 % | 9 | 11.4–137.3 |
|  | 5 km | None | - |
|  | 10 km | 2 | 5.2 – 9.9 |
| 60 - 69 | 5 %, 10 %, 5 km, 10 km | None | - |
| 70 - 79 | 5 % | 3 | 28.1 – 32.2 |
|  | 10 % | 6 | 28.1 – 77.8 |
|  | 5 km | None | - |
|  | 10 km | 1 | 9.4 |
| ≥ 80 | 5 % | 4 | 7.3 – 39.7 |
|  | 10 % | 3 | 6.3 – 173.5 |
|  | 5 km | 2 | 1.4 – 5.0 |
|  | 10 km | 3 | 6.3 – 9.8 |
