# Supplementary material for: Geographical variation and clustering are found in atrial fibrillation beyond socioeconomic differences: a Danish cohort study, 1987–2015
Source: Int J Health Geogr. 2021 Mar 1;20:11. doi: 10.1186/s12942-021-00264-2 (PMC7923319; doi:10.1186/s12942-021-00264-2)

**Additional File 5**

Additional File 5 for article “Geographical variation and clustering are found in atrial fibrillation beyond socio-economic differences: A Danish cohort study, 1987-2015”

Additional file 5: Sensitivity analysis and supplementary analyses.

**Sensitivity analysis - Changed log-gamma parameters**

Geographical variation in residual incidence rate ratios of atrial fibrillation (including atrial flutter) at municipality level compared with country mean (2011-2015) for the fully adjusted model, adjusted for age, sex, socioeconomic position and random effects of municipalities. Maps are shown for two different log-gamma distributions. Municipalities where the residual AF IR is not significantly different from the country mean (i.e. the 95% credibility interval include 1) are grey shaded Map contain data from © EuroGeographics for the administrative boundaries and from The Danish Agency for Data Supply and Efficiency, municipality boarders, 2019.


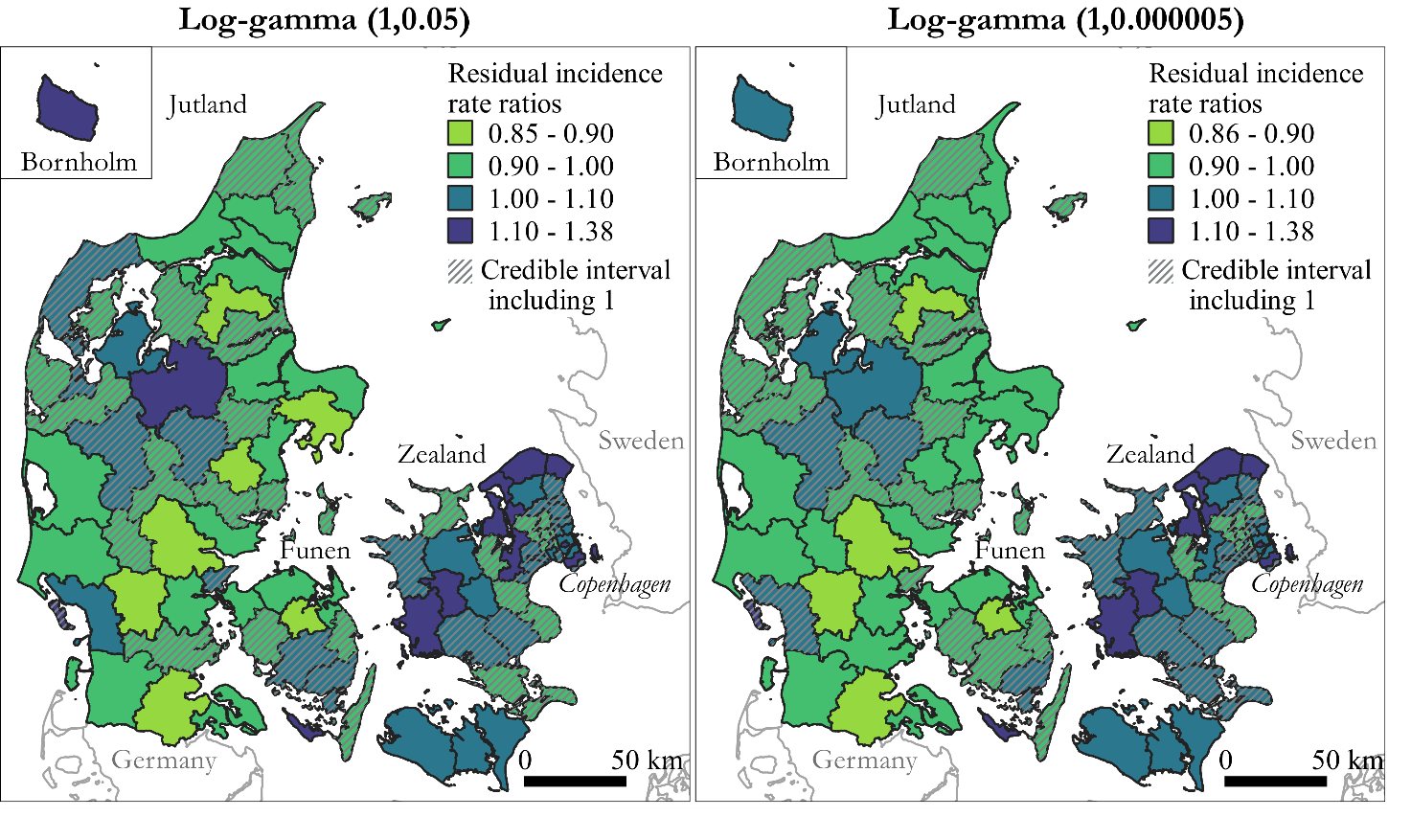


**Supplementary analysis 1 - Pre-2007 municipalities**

Geographical variation in residual incidence rate ratios of atrial fibrillation (including atrial flutter) at municipality level compared with country mean (2011-2015) for the fully adjusted model, adjusted for age, sex, socioeconomic position and random effects of municipalities using municipalities as organized pre-2007 in Denmark (n=275). Municipalities where the residual AF IR is not significantly different from the country mean (i.e. the 95% credibility interval include 1) are grey shaded Map contain data from © EuroGeographics for the administrative boundaries and from The Danish Agency for Data Supply and Efficiency, municipality boarders, 2019.


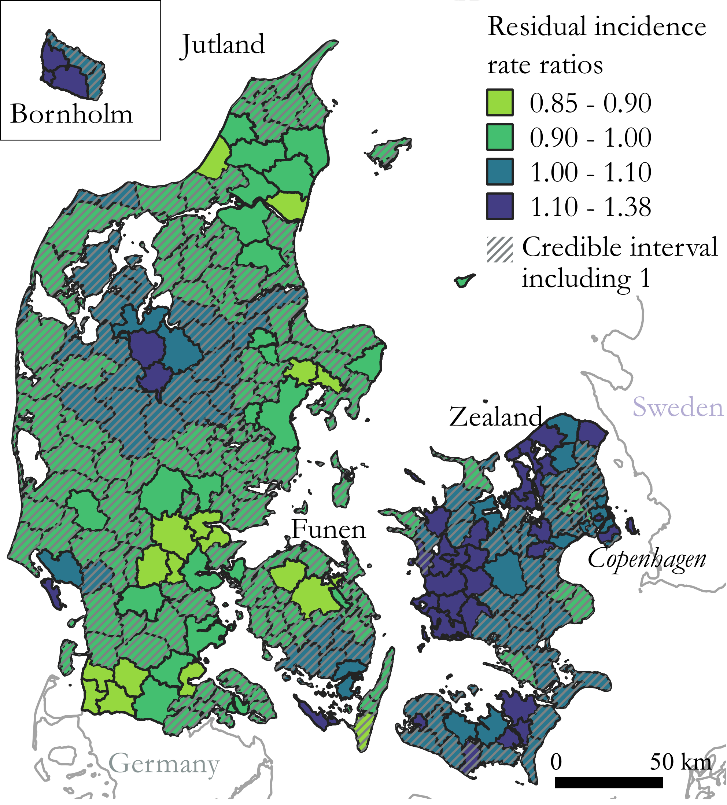


**Supplementary analysis 2 - Only individuals with registered educational level**

Geographical variation in residual incidence rate ratios of atrial fibrillation (including atrial flutter) at municipality level compared with country mean (2011-2015) for the fully adjusted model only including individuals with registered education. The model is adjusted for age, sex, socioeconomic position and random effects of municipalities. Maps are shown for two different log-gamma distributions. Municipalities where the residual AF IR is not significantly different from the country mean (i.e. the 95% credibility interval include 1) are grey shaded Map contain data from © EuroGeographics for the administrative boundaries and from The Danish Agency for Data Supply and Efficiency, municipality boarders, 2019.

.
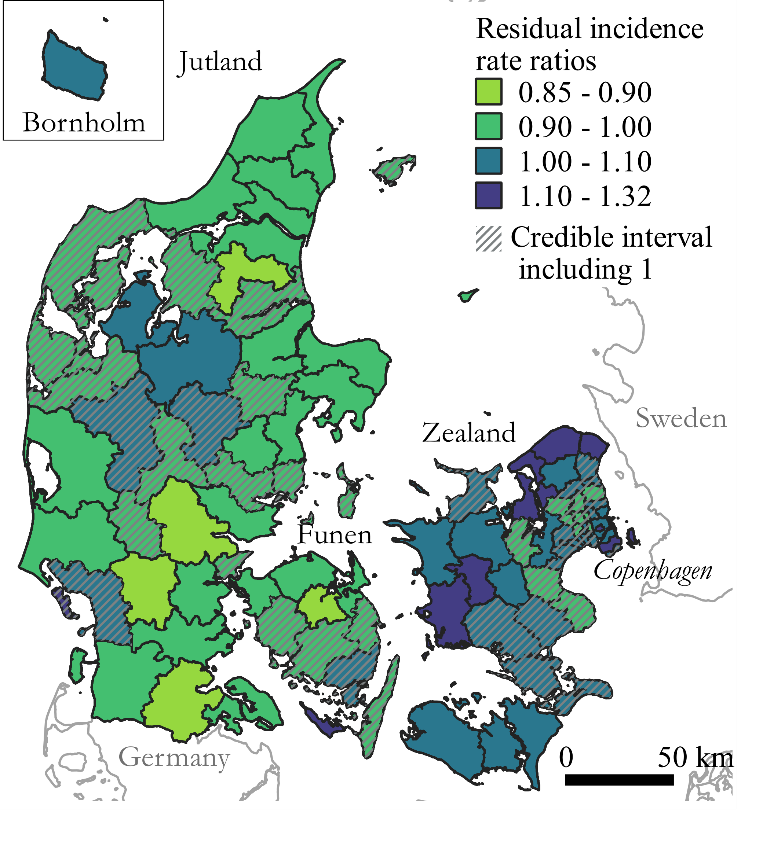


**Supplementary analysis 3 - Further adjustment of hypertension**

Geographical variation in residual incidence rate ratios of atrial fibrillation (including atrial flutter) at municipality level compared with country mean (2011-2015) for the fully adjusted model with further adjusting for hypertension. The model is adjusted for age, sex, socioeconomic position, hypertension and random effects of municipalities. Maps are shown for two different log-gamma distributions. Municipalities where the residual AF IR is not significantly different from the country mean (i.e. the 95% credibility interval include 1) are grey shaded Map contain data from © EuroGeographics for the administrative boundaries and from The Danish Agency for Data Supply and Efficiency, municipality boarders, 2019.


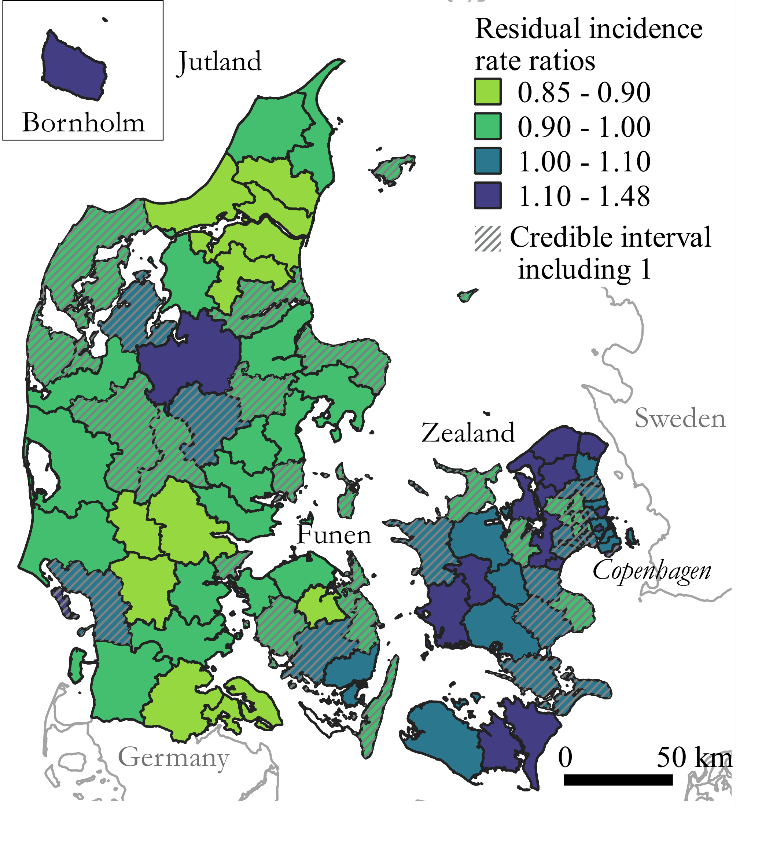

Supplement: Supplementary file 5 — Additional file 5. Sensitivity analysis and supplementary analyses. [file 12942_2021_264_MOESM5_ESM.docx]
